# Associations of Antiphospholipid Antibodies With Splanchnic Vein Thrombosis: A Systematic Review With Meta-Analysis

**DOI:** 10.1097/MD.0000000000000496

**Published:** 2015-01-30

**Authors:** Xingshun Qi, Valerio De Stefano, Chunping Su, Ming Bai, Xiaozhong Guo, Daiming Fan

**Affiliations:** From the Department of Gastroenterology (XQ, XG), General Hospital of Shenyang Military Area, Shenyang; Xijing Hospital of Digestive Diseases (XQ, MB, DF), Fourth Military Medical University, Xi’an, China; Institute of Hematology (VDS), Catholic University, Rome, Italy; and Library of Fourth Military Medical University (CS), Xi’an, China.

## Abstract

Splanchnic vein thrombosis (SVT) refers to Budd–Chiari syndrome (BCS) and portal vein system thrombosis (PVST). Current practice guidelines have recommended the routine screening for antiphospholipid antibodies (APAs) in patients with SVT.

A systematic review and meta-analysis of observational studies was performed to explore the association between APAs and SVT.

The PubMed, EMBASE, and ScienceDirect databases were searched for all relevant papers, in which the prevalence of positive APAs or levels of APAs should be compared between BCS or noncirrhotic PVST patients versus healthy controls, or between cirrhotic patients with portal vein thrombosis (PVT) versus those without PVT.

Fourteen studies were eligible. Only 1 study evaluated the role of APAs in BCS patients and found that positive immunoglobulin (Ig) G anticardiolipin antibody (aCL) was more frequently observed in BCS patients than in healthy controls; however, the associations of other APAs with BCS were not evaluated. Positive IgG aCL was more frequently observed in noncirrhotic patients with PVST than in healthy controls; however, other APAs, such as IgM aCL, lupus anticoagulants (LAs), anti-β_2_-glycoprotein-I antibody (aβ_2_GPI), and aβ_2_GPI-oxidized low-density lipoprotein antibody (ox-LDL) were not associated with noncirrhotic PVST. Positive unclassified aCL was more frequently observed in cirrhotic patients with PVT than in those without PVT; however, the association of IgG aCL and IgM aCL with the development of PVT in liver cirrhosis remained inconsistent among studies.

The risk of BCS and noncirrhotic PVST might be increased by positive IgG aCL but not IgM aCL, LA, aβ_2_GPI, or aβ_2_GPI ox-LDL. However, the evidence regarding APAs in BCS originated from only 1 study. The association between APAs and PVT in liver cirrhosis was unclear.

## Abstract 2

Supplemental Digital Content is available in the text

## INTRODUCTION

Splanchnic vein thrombosis (SVT) consists of Budd–Chiari syndrome (BCS) and portal venous system thrombosis (PVST).^1,2^ The former is characterized by the hepatic venous outflow obstruction after the exclusion of sinusoidal obstructive syndrome. The latter is further classified as portal vein thrombosis (PVT), mesenteric vein thrombosis, and splenic vein thrombosis. Currently, the practice guideline regarding the vascular disorders of the liver has recommended that several thrombotic risk factors should be routinely screened in SVT patients.^3,4^ Antiphospholipid syndrome is regarded as one of the widely accepted thrombotic risk factors, which is defined as a classical triad of arterial and/or venous thrombosis, recurrent fetal loss, and thrombocytopenia in the presence of antiphospholipid antibodies (APAs).^5,6^ APAs primarily include lupus anticoagulant (LA), anticardiolipin antibody (aCL), anti-β_2_-glycoprotein-I antibody (aβ_2_GPI), antiprothrombin, antiphosphatidyl serine, and antiphosphatidyl ethanolamine. Previous systematic reviews have confirmed that these antibodies themselves may be strongly related to the development of thrombotic events within the usual sites.^7–11^ Notably, the highest risks of thrombosis are associated with LA and immunoglobulin (Ig) G aCL/aβ_2_GPI isotype and with an antibody profile including triple positivity for LA, aCL, and aβ_2_GPI.^12–14^ Herein, we performed a systematic review and meta-analysis of observational studies to explore the associations between APAs and SVT.

## METHODS

### Search Strategy

The PubMed, EMBASE, and ScienceDirect databases were searched for the relevant papers. The search items are listed in the Appendix. The last search was performed on January 7, 2014.

### Eligibility Criteria

Eligibility criteria were as follows: the type of papers should be clinical studies but not reviews, comments, or basic studies; the sample size should be ≥10; the participants should be diagnosed with SVT with or without liver cirrhosis; the participants with hepatocellular carcinoma (HCC) should be excluded, because SVT might be attributed to the tumor invasion in HCC; if the case group was BCS or noncirrhotic patients with PVST, the control group should be healthy subjects; if the case group was cirrhotic patients with SVT, the control group should be cirrhotic patients without SVT; the APAs should be detected in both case and control groups; the publication language and form were not limited. If the data were overlapped among 2 or more studies by the same study team, we extracted the data from 1 study with a larger sample size and/or a longer enrollment period.

### Data Extraction

The following data were extracted: first author, publication journal, publication year, country, enrollment period, eligibility criteria, total number of cases and controls, age, sex, methods of APA measurement, proportion of positive APAs in case and control groups, cutoff values for positive APAs, and levels of APAs in case and control groups.

### Study Quality

The study quality was scored by the Newcastle–Ottawa scale, including selection, comparability, and outcome categories. Based on the Newcastle–Ottawa scale, a study can be awarded a maximum of 9 points. Studies with scores of 5 points or more were considered to be of high quality.

### Data Synthesis

Continuous data were evaluated by a mean difference with 95% confidence interval (CI). Then, the mean difference of each study was combined to give a pooled mean difference. Dichotomous data were evaluated by an odds ratio (OR) with 95% CI. Then, the OR of each study was combined to give a pooled OR. A *P* value of <0.05 was considered statistically significant for the effect size. Data were pooled by using a random-effects model. Heterogeneity between studies was assessed by using the *I*^2^ statistic (*I*^2^ > 50% was considered as having substantial heterogeneity) and the χ^2^ test (*P* < 0.10 was considered to represent significant statistical heterogeneity). All analyses were conducted using the statistical package Review Manager version 5.2 (Copenhagen, The Nordic Cochrane Center, The Cochrane Collaboration, 2011).

## RESULTS

### Study Selection

**FIGURE 1:**
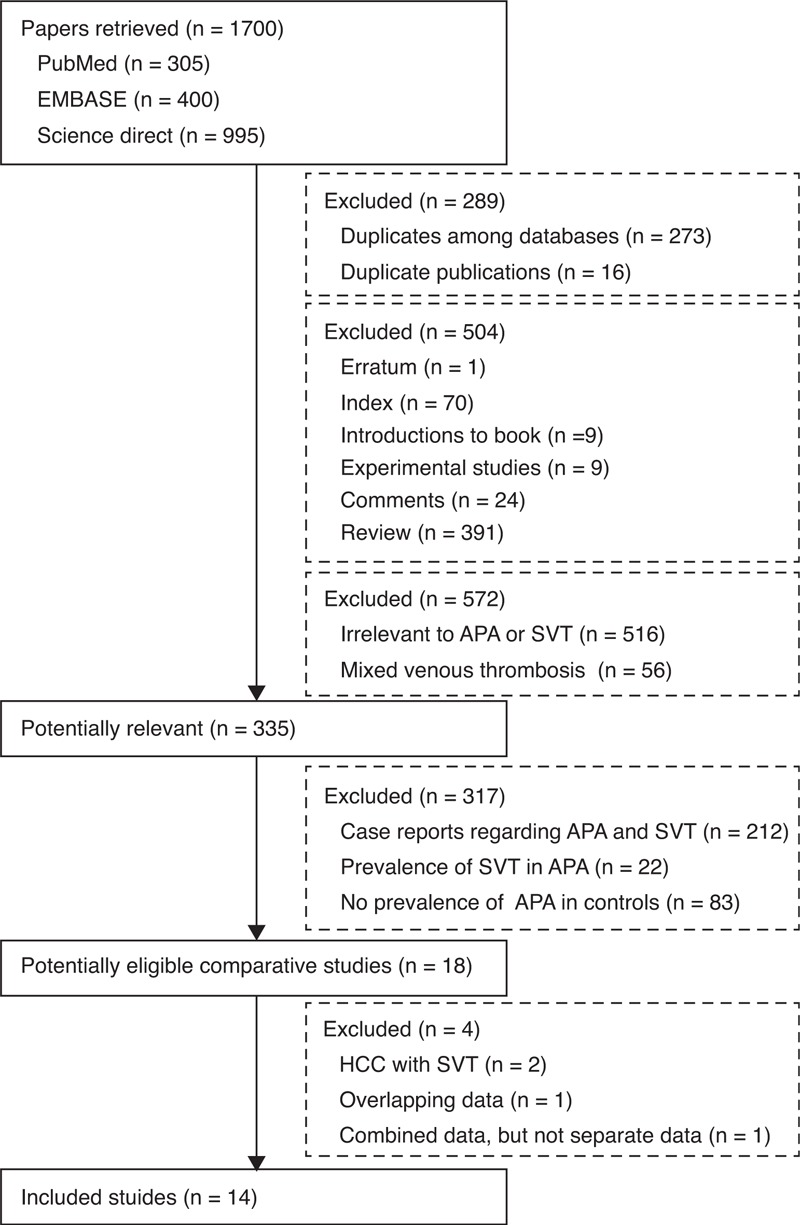
Flowchart of study inclusion. APA = antiphospholipid antibody, HCC = hepatocellular carcinoma, SVT = splanchnic vein thrombosis.

Overall, 1700 papers were retrieved via the 3 databases. Among them, 18 studies were eligible. However, 4 studies were further excluded, because the levels of APAs were reported in SVT patients with HCC in 2 studies,^15,16^ the enrollment period was shorter in 1 study,^17^ and only the combined data regarding the biological antiphospholipid syndrome (aCLs and LA) were given in 1 study.^18^ Thus, 14 studies were finally included in the systematic review^19–32^ (Figure 1). Notably, 5 studies conducted by the same study team were included,^20–24^ because the APA tests, types of patients, and/or enrollment periods were different among them.

### Study Characteristics

The characteristics of these included studies were summarized in Table 1. All included studies were conducted in Europe and Asia, including Italy (n = 8), Turkey (n = 3), India (n = 2), and Spain (n = 1). One study enrolled BCS patients,^19^ 5 studies enrolled noncirrhotic patients with PVST alone,^22,24–26,31^ 7 studies enrolled cirrhotic patients with PVT alone,^21,23,27–30,32^ and 1 study enrolled both cirrhotic and noncirrhotic patients with PVT.^20^ Eligibility criteria and methods of APA measurement were summarized in Supplementary Tables 1 and 2, http://links.lww.com/MD/A196, respectively.

**TABLE 1:**
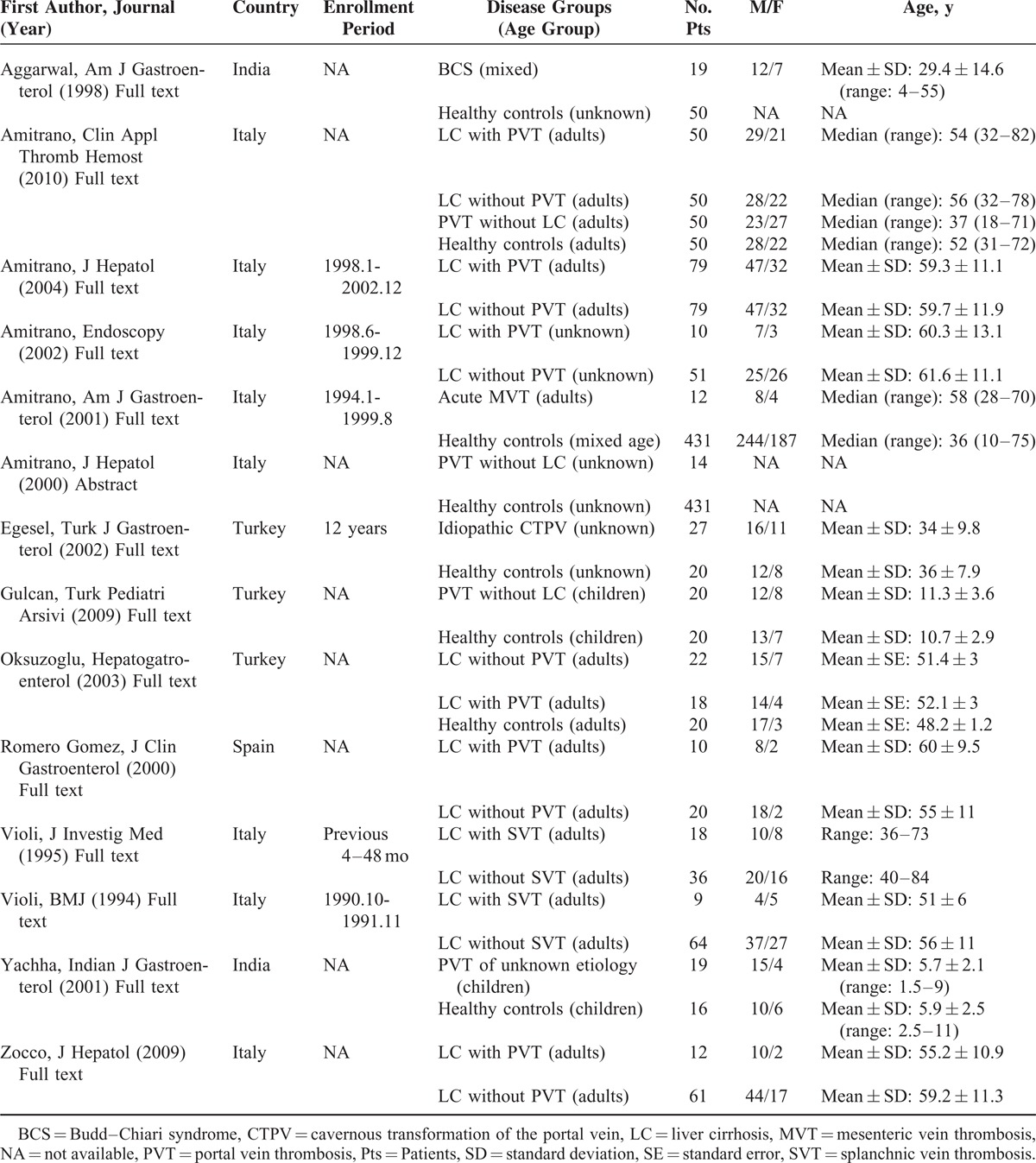
Characteristics of Included Studies

### Study Quality

Of these included studies, 13 were considered to be of relatively high quality (Supplementary Table 3, http://links.lww.com/MD/A196).

### Meta-Analyses

The relevant data from every included study were summarized in Supplementary Tables 4–22, http://links.lww.com/MD/A196. Results of systematic reviews and meta-analyses were summarized in Table 2.

**TABLE 2:**
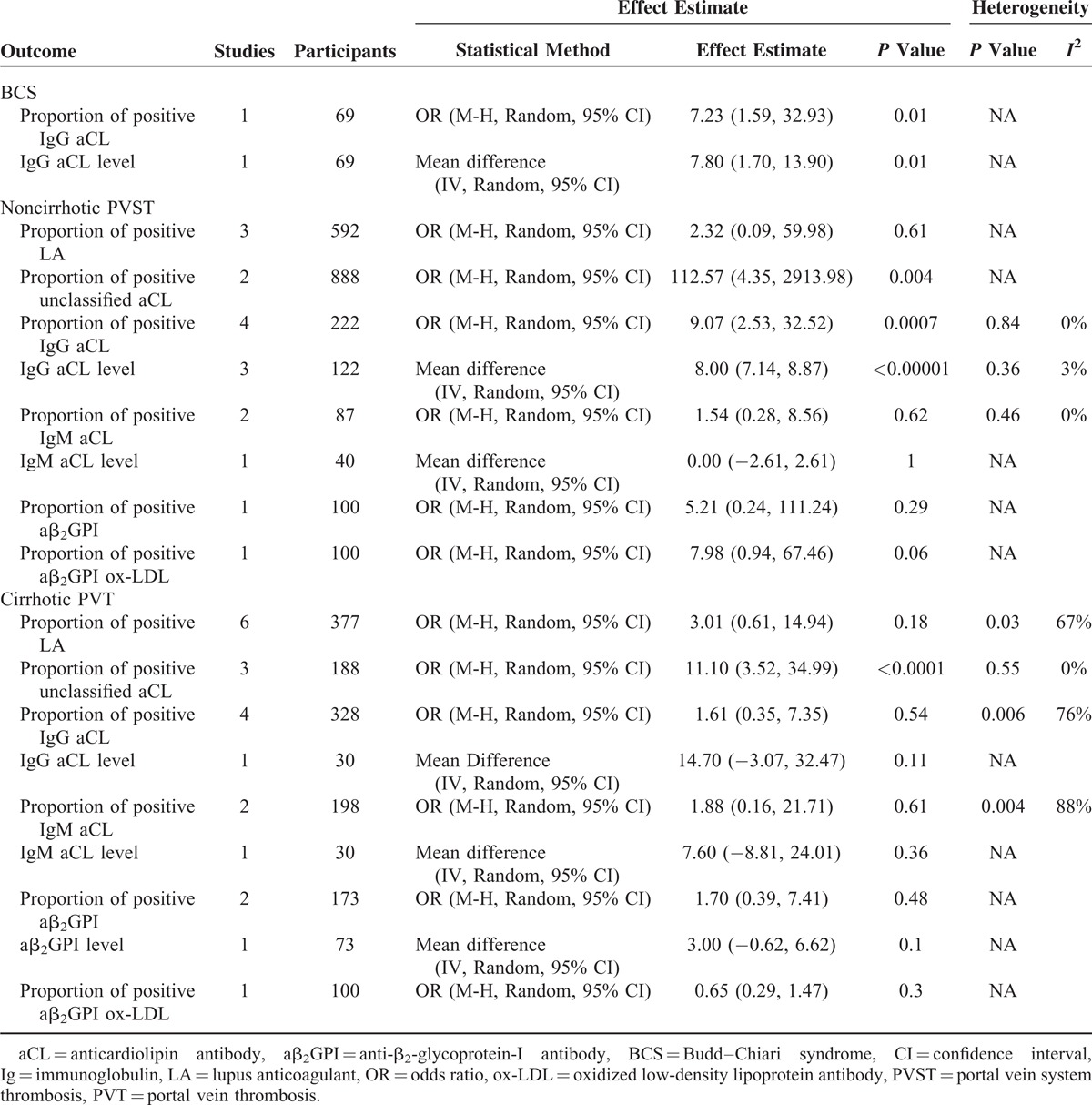
Results of Meta-Analyses

### Budd–Chiari Syndrome

#### Immunoglobulin G aCL

BCS patients were investigated in only 1 study, and had a significantly higher proportion of positive IgG aCL or IgG aCL level than healthy controls.^19^

### Noncirrhotic PVST

#### Lupus Anticoagulant

Meta-analysis of 3 studies demonstrated that the proportion of positive LA was not significantly different between noncirrhotic patients with PVST and healthy controls.^20,24,25^ Notably, 2 of them showed that the prevalence of positive LA was 0 in either noncirrhotic patients with PVST or healthy controls.^20,24^

#### Unclassified aCL

Meta-analysis of 2 studies demonstrated that the proportion of positive unclassified aCL was significantly higher in noncirrhotic patients with PVST than in healthy controls.^22,24^ Notably, 1 of them showed that the prevalence of positive unclassified aCL was 0 in either noncirrhotic patients with PVST or healthy controls.^24^

#### Immunoglobulin G aCL

**FIGURE 2:**
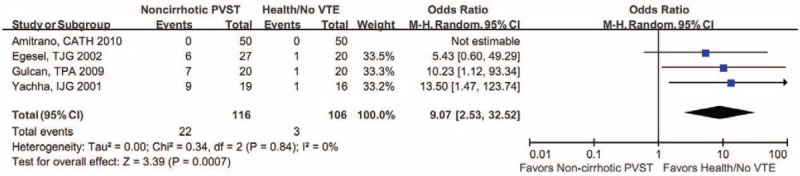
Forest plot comparing the proportion of positive IgG aCL between noncirrhotic patients with PVST and healthy controls without venous thromboembolism. aCL = anticardiolipin antibody, CI = confidence interval, Ig = immunoglobulin, PVST = portal vein system thrombosis, VTE = venous thromboembolism.

Meta-analysis of 4 studies demonstrated that the proportion of positive IgG aCL was significantly higher in noncirrhotic patients with PVST than in healthy controls (Figure 2).^20,25,26,31^ Notably, 1 of them showed that the prevalence of positive IgG aCL was 0 in either noncirrhotic patients with PVST or healthy controls.^20^ In addition, meta-analysis of 3 studies demonstrated that the IgG aCL level was significantly higher in noncirrhotic patients with PVST than in healthy controls.^25,26,31^

#### Immunoglobulin M aCL

Meta-analysis of 2 studies demonstrated that the proportion of positive IgM aCL was not significantly different between noncirrhotic patients with PVST and healthy controls.^25,26^ In addition, 1 study demonstrated that the IgG aCL level was not significantly different between the 2 groups.^26^

#### Anti-β_2_-Glycoprotein-I Antibody

Only 1 study demonstrated that the proportion of positive aβ_2_GPI was not significantly different between noncirrhotic patients with PVST and healthy controls.^20^

#### aβ_2_GPI-Oxidized Low-Density Lipoprotein Antibody

Only 1 study demonstrated that the proportion of positive aβ_2_GPI-oxidized low-density lipoprotein antibody (ox-LDL) was not significantly different between noncirrhotic patients with PVST and healthy controls.^20^

### Cirrhotic PVT

#### Lupus Anticoagulant

**FIGURE 3:**
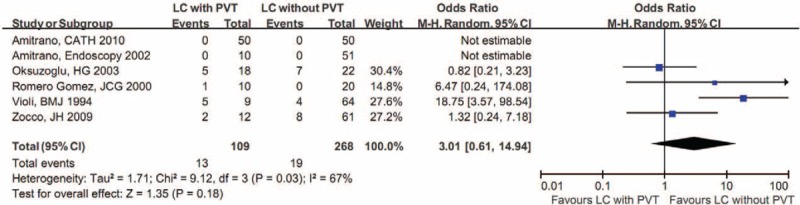
Forest plot comparing the proportion of positive LA between cirrhotic patients with and without PVT. CI = confidence interval, LA = lupus anticoagulant, LC = liver cirrhosis, PVT = portal vein thrombosis.

Meta-analysis of 6 studies demonstrated that the proportion of positive LA was not significantly different between cirrhotic patients with and without PVT (Figure 3).^20,23,27,28,30,32^ Notably, 2 of them showed that the prevalence of positive LA was 0 in both cirrhotic patients with and without PVT.^20,23^

#### Unclassified aCL

Meta-analysis of 3 studies demonstrated that the proportion of positive unclassified aCL was significantly higher in cirrhotic patients with PVT than in those without PVT.^23,29,30^

#### Immunoglobulin G aCL

**FIGURE 4:**
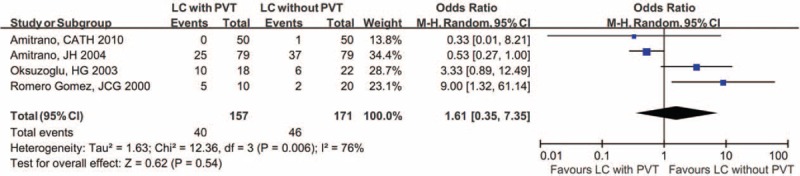
Forest plot comparing the proportion of positive IgG aCL between cirrhotic patients with and without PVT. aCL = anticardiolipin antibody, CI = confidence interval, IgG = immunoglobulin G, LC = liver cirrhosis, PVT = portal vein thrombosis.

Meta-analysis of 4 studies demonstrated that the proportion of positive IgG aCL was not significantly different between cirrhotic patients with and without PVT (Figure 4).^20,23,27,28^ In addition, the IgG aCL level was expressed as mean with standard deviation in 1 study,^28^ and as median with interquartile ratio in another study.^27^ Therefore, a meta-analysis regarding IgG aCL level could not be performed. In details, the former study reported that the IgG aCL level was not significantly different between the 2 groups,^28^ but the latter study found that IgG aCL level was significantly higher in cirrhotic patients with PVT than in those without PVT (*P* = 0.014).^27^

#### Immunoglobulin M aCL

Meta-analysis of 2 studies demonstrated that the proportion of positive IgM aCL was not significantly different between cirrhotic patients with and without PVT.^21,27^ In addition, the IgM aCL level was expressed as mean with standard deviation in 1 study^28^ and as median with interquartile ratio in another study.^27^ Therefore, a meta-analysis regarding IgM aCL level could not be performed. In details, the former study reported that the IgM aCL level was not significantly different between the 2 groups,^28^ but the latter study found that the IgM aCL level was significantly higher in cirrhotic patients with PVT than in those without PVT (*P* = 0.001).^27^

#### Anti-β_2_-Glycoprotein-I Antibody

Meta-analysis of 2 studies demonstrated that the proportion of positive aβ_2_GPI was not significantly different between cirrhotic patients with and without PVT.^20,32^ Notably, 1 of them demonstrated that the prevalence of positive aβ_2_GPI was 0 in both cirrhotic patients with and without PVT.^20^ In addition, 1 study demonstrated that the aβ_2_GPI level was not significantly different between the 2 groups.^32^

#### Anti-β_2_-Glycoprotein-I Antibody ox-LDL

Only 1 study demonstrated that the proportion of positive aβ_2_GPI ox-LDL was not significantly different between cirrhotic patients with and without PVT.^20^

## DISCUSSION

Our previous works have systematically evaluated the role of several thrombotic risk factors in the development of SVT, including JAK2 V617F mutation, inherited antithrombin, protein C and protein S deficiencies, factor V Leiden and prothrombin G20210A mutation, methylenetetrahydrofolate reductase C677T mutation, and hyperhomocysteinemia.^33–36^ The present systematic review has for the first time collected all available evidence regarding the associations between APAs and SVT. The important findings were as follows. First, IgG aCL was positively associated with BCS. Second, unclassified aCL was positively associated with the development of PVST in noncirrhotic patients; and this positive association was attributed to IgG type but not IgM type. Third, LA, aβ_2_GPI, and aβ_2_GPI ox-LDL were not associated with the development of PVST in noncirrhotic patients. Fourth, unclassified aCL was positively associated with the development of PVT in cirrhotic patients, but this positive association could not be achieved in the meta-analyses regarding IgG or IgM aCL. Fifth, LA, aβ_2_GPI, and aβ_2_GPI ox-LDL were not associated with the development of PVT in cirrhotic patients.

On the basis of an association of IgG aCL with BCS and noncirrhotic PVST, the routine screening for IgG aCL should be recommended. However, the relevant data were very limited in BCS patients, which might influence the reproducibility of our conclusion. Additionally, we would like to emphasize that the significance of other APAs in the pathogenesis of BCS and noncirrhotic PVST should be greatly toned down. Accordingly, the screening tests for LA and IgM aCL might be unnecessary in such patients.

Despite a positive association between unclassified aCL and PVT in liver cirrhosis, we did not establish any positive associations of IgG aCL or IgM aCL with PVT. To explain the unexpected phenomenon, we rechecked the data from every individual study. In the meta-analysis regarding unclassified aCL, all of the 3 included studies demonstrated a higher incidence of positive unclassified aCL in cirrhotic patients with PVT.^23,29,30^ By comparison, in the meta-analysis regarding IgG aCL, 2 of the 4 included studies demonstrated a higher proportion of positive IgG aCL in cirrhotic patients with PVT,^27,28^ and another 2 studies with a relatively larger sample size achieved the opposite results.^20,21^ Furthermore, the cutoffs for positive IgG aCL were close (20 U/mL or 23 GPI units) in the former 2 studies^27,28^ but very different (10 U/mL or 40 GPI units) in the latter 2 studies.^20,21^ It should be noted that either an underestimated or overestimated cutoff might result in the reporting bias. In the study with a cutoff of 10 U/mL, 32% of cirrhotic patients with PVT had a positive IgG aCL, and 47% of cirrhotic patients without PVT had a positive IgG aCL.^21^ By contrast, in the study with a cutoff of 40 GPL units, none of cirrhotic patients with PVT had a positive IgG aCL, and only 2% of cirrhotic patients without PVT had a positive IgG aCL.^20^ Given the heterogeneous cutoffs among studies, the association needed to be further confirmed in studies with a larger sample size and an appropriate cutoff for positive IgG aCL.

On the other hand, positive APAs could be frequently found in chronic hepatitis virus C infection-related liver diseases without any evidence of venous thrombosis.^37–43^ Positive APAs were regarded as an epiphenomenon of chronic liver injury,^38,42^ which might be produced due to the immunologic disturbances induced by hepatitis C virus infection or prolonged tissue damage in systemic organs.^43^ Biron et al^38^ also found that the proportion of positive APAs was positively associated with the severity of liver dysfunction. Certainly, we arbitrarily selected liver cirrhosis without PVT as the control group to balance the potential bias caused by the presence of liver diseases.

The major limitation of this study was that evidence concerning BCS patients is restricted to only 1 study, and that a relatively small number of studies concerning PVST were included in every meta-analysis, especially in the meta-analyses regarding aβ_2_GPI and aβ_2_GPI ox-LDL. All included studies had a small sample size. In addition, no study investigated the presence of triple-positive APA profiles, which is relevant in the development of the thrombotic risk.^12–14^ Moreover, we had to acknowledge that that our search strategy was extensive via the 3 major databases. This suggested the necessity of further validation studies in this field.

In conclusion, based on the currently available evidence, IgG aCL was positively associated with the development of BCS and noncirrhotic PVST. However, other APAs might not be considered as the potential thrombotic risk factors for BCS and noncirrhotic PVST. Notably, given that the evidence regarding APAs in BCS originated from only 1 study, the conclusion should be confirmed in more studies. The association between aCL and the development of PVT in liver cirrhosis needed to be further explored.

## Appendix

**Search #1:** (portal vein thrombosis) **OR** (portal venous thrombosis) **OR** (portal vein thrombus) **OR** (portal venous thrombus) **OR** (portal vein obstruction) **OR** (portal venous obstruction) **OR** (portal vein occlusion) **OR** (portal venous occlusion) **OR** (thrombotic portal vein) **OR** (thrombosed portal vein) **OR** (occluded portal vein) **OR** (occlusive portal vein) **OR** (obstructed portal vein) **OR** (obstructive portal vein) **OR** (portal cavernoma) **OR** (cavernous transformation of portal vein) **OR** (Budd Chiari) **OR** (hepatic vein thrombosis) **OR** (hepatic venous thrombosis) **OR** (hepatic vein obstruction) **OR** (hepatic venous obstruction) **OR** (hepatic vein occlusion) **OR** (hepatic venous occlusion) **OR** (mesenteric vein thrombosis) **OR** (mesenteric venous thrombosis) **OR** (mesenteric vein obstruction) **OR** (mesenteric venous obstruction) **OR** (mesenteric vein occlusion) **OR** (mesenteric venous occlusion) **OR** (splenic vein thrombosis) **OR** (splenic venous thrombosis) **OR** (splenic vein obstruction) **OR** (splenic venous obstruction) **OR** (splenic vein occlusion) **OR** (splenic venous occlusion) **OR** (splanchnic vein thrombosis) **OR** (splanchnic venous thrombosis) **OR** (splanchnic vein obstruction) **OR** (splanchnic venous obstruction) **OR** (splanchnic vein occlusion) **OR** (splanchnic venous occlusion) **OR** (abdominal vein thrombosis) **OR** (abdominal venous thrombosis) **OR** (abdominal vein obstruction) **OR** (abdominal venous obstruction) **OR** (abdominal vein occlusion) **OR** (abdominal venous occlusion) **OR (**portosplenomesenteric vein thrombosis) **OR** (portosplenomesenteric venous thrombosis) **OR** (portosplenomesenteric vein occlusion) **OR** (portosplenomesenteric venous occlusion) **OR** (portosplenomesenteric vein obstruction) **OR** (portosplenomesenteric venous obstruction)

**Search #2:** (antiphospholipid) **OR** (anticardiolipin) **OR** (lupus anticoagulant) **OR** (anti-β2-glycoprotein I) **OR** (anti-beta2-glycoprotein I) **OR** (anti-phosphatidylcholine) **OR** (anti-phosphatidylethanolamine) **OR** (anti-phosphatidylinositol) **OR** (anti-phosphatidylserine) **OR** (anti-sphingomyeline)

Search #3: #1 AND #2
